# Glucocorticoid Receptor and SUMO Fluctuations in Response to Pulsatile Glucocorticoids In Vitro and in Male Rat Brains

**DOI:** 10.1210/endocr/bqaf140

**Published:** 2025-09-17

**Authors:** Caroline A Rivers, Heyam A Abdulqayoom, Yvonne M Kershaw, Oliver R Troy, Zidong Zhao, Becky L Conway-Campbell, Stafford L Lightman

**Affiliations:** Bristol Medical School, Translational Health Science, University of Bristol, Bristol BS1 3NY, UK; Bristol Medical School, Translational Health Science, University of Bristol, Bristol BS1 3NY, UK; Bristol Medical School, Translational Health Science, University of Bristol, Bristol BS1 3NY, UK; Bristol Medical School, Translational Health Science, University of Bristol, Bristol BS1 3NY, UK; Bristol Medical School, Translational Health Science, University of Bristol, Bristol BS1 3NY, UK; Bristol Medical School, Translational Health Science, University of Bristol, Bristol BS1 3NY, UK; Bristol Medical School, Translational Health Science, University of Bristol, Bristol BS1 3NY, UK

**Keywords:** glucocorticoid, receptor, SUMOylation, pulsatile, hormone

## Abstract

We present a molecular mechanism underpinning how pulsatile patterns of glucocorticoid hormones maintain signal responsivity, evade hormone resistance, and promote homeostasis. Endogenous glucocorticoids are released in a pulsatile manner resulting in oscillating hormone signals with intermittent peaks of high glucocorticoids and troughs of low glucocorticoids. We show that ligand activation of glucocorticoid receptors rapidly triggers the post-translational modification SUMOylation, which is coupled to receptor degradation, whereby resistance to subsequent signal transduction is generated and ligand response attenuated. We find rapid, transient glucocorticoid receptor SUMOylation tracks ultradian (roughly hourly) pulse dynamics in cells, as well as circadian (daily) oscillatory rhythms in vivo, enabling cellular interpretation of fluctuating hormone patterns. Prolonged treatment with the long-acting synthetic glucocorticoid methylprednisolone disrupted glucocorticoid receptor SUMOylation levels in rat brain tissue. Pharmacological glucocorticoid therapy generates unremitting glucocorticoid signaling, which may substantially reduce the glucocorticoid receptor pool and contribute to the therapeutic problem of acquired glucocorticoid resistance. The physiological solution for maintaining signal responsivity over time is pulsatile hormone exposure, with pulsatile low glucocorticoid troughs which periodically limit receptor degradation and associated signal attenuation. We show low glucocorticoid periods allow time for depleted glucocorticoid receptor expression levels to recover and thereby maintain signal sensitivity. Our results reveal a molecular mechanism responsive to hormone pattern information, through which endogenous ultradian and circadian glucocorticoid fluctuations maintain glucocorticoid receptor expression and glucocorticoid sensitivity. Dynamic ligand-activated glucocorticoid receptor SUMOylation coupled to degradation is revealed as a component of glucocorticoid receptor protein regulation, whose expression is critical for metabolic, immunological, cognitive, and cardiovascular homeostasis.

Glucocorticoid hormones (GCs) maintain homeostasis in response to diurnal changes and stressful perturbations through metabolic, immunological, cardiovascular, and cognitive regulation. Cellular sensitivity to GCs depends on several local factors—the availability of GCs, GC metabolism, and the expression of transcriptional and translational isoforms of the glucocorticoid receptor (GR) (1) and mineralocorticoid receptor. The predominant GR form is GRα which is widely expressed; GRβ is less abundant and impairs GRα-mediated activities (2) but also has specific direct effects of its own (3-5).

The chronic presence of GCs ultimately leads to acquired GC resistance, which remains an unsolved problem for GC therapy and chronic inflammatory conditions. GCs cause GRα downregulation, which both reduces GRα availability and increases the relative proportion of GRβ to oppose GRα activities (2, 6). GCs dramatically reduce the half-life of GRα, from 70 hours for unliganded GRα to 22 hours with the endogenous ligand cortisol (3). Ligand activation confers enhanced dimer stability, as GRs bind to DNA at classic palindromic GC response elements and induce RNA transcription (7). Dimerization-promoting GCs such as dexamethasone and cortisol increase GRα turnover while dimerization-abrogating compound A minimizes turnover (3). Thus ligand activation, GR dimerization, RNA transcription, and GR turnover are closely coupled.

GCs enhance GRα protein turnover via the ubiquitin-proteasome system (8). Transient transcription factors are commonly found to activate target genes concomitant with their own degradation through the ubiquitin-proteasome system (9). This coupling of transcription factor activity with transcription factor destruction is a fundamentally important conserved cellular process that limits uncontrolled signal activation.

Crosstalk exists between the SUMOylation and ubiquitin-proteasomal pathways (10). SUMOylation can function as a secondary signal mediating ubiquitin-dependent degradation by the proteasome (11, 12) and inhibition of ubiquitinylation causes accumulation of SUMO-modified nuclear substrates, including SUMOylated transcription factors (13). Protein SUMOylation promotes liquid/liquid phase-separation facilitating the rapid formation of molecular condensates with increased local concentrations of factors (14, 15). The functions of SUMOylated proteins include gene transcription regulation (16), chromatin remodeling (17), development (18), circadian rhythm (19), and the response to inflammation (20). Transcription factor SUMOylation is often associated with limiting transcriptional activation (21-23), although transcription factor SUMOylation can also facilitate repressor recruitment (24).

The GR has 3 consensus SUMOylation sites (25). RWD-containing SUMOylation enhancer (RSUME), whose expression increases in stress conditions such as heat shock, promotes GR SUMOylation at the ligand binding domain site and increases GR transcriptional activity (26). SUMOylation of the 2 N-terminal sites enhances transcriptional activity but limits synergistic transactivation on promoters with closely spaced GR binding sites (25, 27). At gene promoters, promoter-bound basal transcription machinery is regulated by dynamic SUMOylation (28). Since SUMO pathway enzymes have low specificity (29), recruitment of SUMO-ligases to promoters can cause localized proteins to be SUMOylated (30), so that the chromatin-association of specific transcription factors may lead to their dynamic SUMOylation.

Analysis of the protein network around chromatin-associated GR showed preferential interaction of SUMO-competent GR (GR-wt) with proteins implicated in transcription repression; while SUMO-deficient GR (GR3KR) was more potent in binding and opening chromatin at GC-regulated enhancers and inducing transcription (31). Thus, GR SUMOylation can limit transcriptional activation and facilitate repressor recruitment. GR autoregulation also occurs, as GR downregulation was promoted by GR SUMOylation in SUMO1 overexpression experiments (32).

We are interested in studying the rapid changes following GC activation as a way into understanding the function of the endogenous GC ultradian rhythm with its dynamic GC signals and downstream gene pulsing (33, 34). Here we investigated GR SUMO modification because SUMOylation is typically rapid and transient; and many transcription factors are dynamically modified by a wave of SUMOylation (23, 35). We found GC-induced GR SUMOylation to be a trigger for GR protein degradation, which subsequently reduces GC signal transduction. We reveal a function for hormone nadirs (low-hormone periods) as time intervals during which GR protein expression can recover so that GC responsivity is restored. We show in vivo GR SUMOylation fluctuates over the circadian rhythm as GC hormone levels oscillate. Overall, we show that the dynamic dimension of GC hormone signaling should be taken into consideration as the pattern of hormone presentation regulates responses, not only a signal's amplitude and integral.

## Materials and Methods

### Cells and Reagents

A549 human lung adenocarcinoma epithelial cells (RRID:CVCL_0023) were purchased from European Collection of Authenticated Cell Cultures (ECACC) and maintained in Dulbecco's Modified Eagle Medium (DMEM) supplemented with 10% fetal bovine serum (Thermo Fisher Scientific, Waltham, USA). The stably GR-expressing Flp-InTM HEK293 cell lines (HEK293-GR-wt and HEK293-GR3KR) (36) were generously donated by V. Paakinaho (Finland) and maintained in DMEM supplemented with 10% fetal bovine serum, 25 U/mL penicillin and 25 µg/mL streptomycin (Thermo Fisher Scientific, Waltham, USA) and 100 µg/mL hygromycin-B (NBS Biologicals, Huntingdon, UK). Cells were used up to passage 20 and routinely tested for mycoplasma. To washout GCs, media was exchanged twice with Dulbecco's phosphate-buffered saline, then with CSS media (DMEM without phenol red supplemented with 10% charcoal-stripped serum). Hydrocortisone, dexamethasone, and cycloheximide were from Sigma (Gillingham, UK); ML792 (HY-108702-5 mg) from Cambridge Bioscience (Cambridge, UK).

### Plasmids

Expression plasmid EGFP-GRα was purchased from GenScript and encoded the open reading frame of human NR3C1 cDNA with N-terminal EGFP tag in pcDNA3.1. The SUMO mutant GRmutSUMO was generated by point mutation of the lysine codons (AAA/AAG) at K277, K293, and K703 to arginine codons (AGA/AGG) by overlapping polymerase chain reaction (PCR) and traditional cloning techniques. The partial mutants GRmutS12 and GRmutS3 were similarly created by overlapping PCR. Plasmid DNA sequences were confirmed by Sanger sequencing.

### Immunoprecipitations and Westerns

Whole cell lysates were prepared with RIPA buffer (Tris 50mM pH 7.5, NaCl 150mM, Nonidet-P-40 1%, sodium deoxycholate 0.5%, EDTA 1mM) with protease inhibitors (cOmplete™ EDTA-free Protease Inhibitors, Roche). Freshly prepared N-ethylmaleimide (NEM) 20mM (#04259 Sigma, Gillingham, UK) was added to inhibit SUMO degradation. Lysates were sonicated for 5 seconds with a Branson probe sonicator, cleared by centrifugation (16 000*g* for 20 minutes at 4 °C) and quantified by BCA assay (23225 Pierce, Rockford, USA).

GFP-Trap immunoprecipitation from cultured cells was performed with 2 million A549 cells in 6-cm dishes reverse transfected with 7.5 µg GFP-GR/GFP-GRmutSUMO plasmid. The entire sample was used for immunoprecipitation, apart from 1% lysate which was removed for input normalization, with 25 µL GFP-Trap magnetic agarose beads (ChromoTek Cat# gtma-20, RRID:AB_2631358). Immunoblots were probed with anti-SUMO-2/3 (18H8) rabbit mAb (Cell Signaling Technology Cat# 4971, RRID:AB_2198425) at 1:1000, followed by anti-GR antibody (Proteintech Cat# 24050-1-AP, RRID:AB_2813890) at 1:5000 to assess levels of SUMOylated and unSUMOylated GFP-GR.

ChromoTek SUMO-Trap magnetic agarose beads (Proteintech Cat# sutma, RRID:AB_3668963) and ChromoTek Ubiquitin-Trap magnetic agarose beads (ChromoTek Cat# utma, RRID:AB_2943578) were used for immunoprecipitations according to the manufacturer’s instructions. For each immunoprecipitation, 1 million A549 GR-HiBiT cells were seeded in 10-cm dishes and whole cell lysates were collected on the following day after hormone treatments. Then 2% lysate was removed to assess input loading, and the remaining lysate was incubated with 25 µL SUMO/Ubiquitin-Trap magnetic agarose beads overnight to pull down SUMOylated and ubiquitinylated proteins. Immunoblots were probed for the 11–amino acid sequence tag *HiBiT* using Nano-Glo® HiBiT Blotting (N2410 Promega, Madison, USA) and imaged with an Azure 600 Imager (Azure Biosystems, Dublin, USA).

Rat brain (rat prefrontal cortex) tissue immunoprecipitation was performed with SUMO-2/3 Affinity Beads (Cytoskeleton Cat# ASM24-Beads, RRID:AB_3712333) to detect endogenous SUMOylated GR. Frozen tissue was lysed in RIPA buffer supplemented with NEM, Dounce-homogenized, sonicated for 5 seconds, cleared by centrifugation, and quantified by BCA assay as for cultured cell samples. Each immunoprecipitation used 2 mg total protein with 40 µL SUMO-2/3 Affinity Beads. Anti-GR antibody (Proteintech Cat# 24050-1-AP, RRID:AB_2813890) at 1:5000 was used to probe immunoblots and 1% lysate samples for input normalization. Densitometry measurements were obtained using ImageJ software.

### Transfection, RNA Extraction, and RT-qPCR

Cells were washed twice in Dulbecco's phosphate-buffered saline to remove hormone, seeded at 0.4 × 10^6^ cells/well in 6-well plates in CSS media, and cultured for 24 hours in the absence of hormone. After hormone treatment, RNA was harvested with Trizol reagent (Invitrogen, Paisley, UK) and precipitated with isopropanol and GlycoBlue at −20 °C overnight. Residual DNA was eliminated by incubation with Turbo DNase. 1 µg total RNA was reverse transcribed with Superscript III (10368252, Invitrogen, Paisley, UK) according to the manufacturer's instructions. Real-time PCR with PowerUp™ SYBR™ Green (Applied Biosystems, Warrington, UK) with standard curve analysis was used to quantify transcript expression. qPCR primers were designed using the tool PrimerQuest (Integrated DNA Technologies, Coralville, USA).

### CrispR Modification

The HiBiT tag sequence was introduced into NR3C1 after the alternatively spliced terminal exon 9α in A549 cells, as previously published (37). crRNA and trRNA (Alt-R CrispR RNA and transactivating crRNA, IDT, Coralville, USA) were annealed to form guide RNA, which was incubated with Cas9 protein (Alt-R S.p. Cas9 Nuclease V3 #1081058, IDT, Coralville, USA) to assemble ribonucleoprotein complexes. Ribonucleoprotein was delivered with single-stranded donor DNA (HDR donor oligonucleotides, IDT, Coralville, USA) to A549 cells by electroporation using Nucleofector Kit T (# VCA-1002, Lonza, Basel, Switzerland). Guide 1: TAAGGCAACCATTCTTATTA; Guide 2: AGTGACTGCCTTAATAAGAA; Donor DNA: TACCAAAATATTCAAATGGAAATATCAAAAAACTTCTGTTTCATCAAAAGGTCTCCGTGAGCGGCTGGCGGCTGTTCAAGAAGATTAGCTGACTGCGTTATTAAGAATCGTTGCCTTAAAGAAAGTCGAATTAATAGCTTTT.

HiBiT-positive clones were selected after limited dilution of CrispR-modified cells. Integration of HiBiT at the 3′ end of NR3C1, upstream of the STOP codon, was confirmed by PCR analysis and Sanger sequencing (Fig. S1 (38)). HiBiT expression was assayed with Nano-Glo® HiBiT Lytic Detection System (N3030 Promega, Madison, USA) and luminescence measured using a GloMax® microplate reader (Promega, Madison, USA).

### Animals and Ethical Approval

Adult male Lister-Hooded rats (220-240 g, Envigo/Inotiv, Mill Hill, UK) were housed with ad libitum access to food and water or 0.9% physiological saline in the case of adrenalectomized (ADX) rats. They were kept under 12:12 light/dark (light on at 08:00 hours) with normal laboratory conditions. We have complied with all relevant ethical regulations for animal testing. All animal procedures were carried out in accordance with the ARRIVE guidelines (39, 40), UK Home Office animal welfare regulations, and approved by written consent from the Animal Welfare and Ethical Review Body (University of Bristol, UK).

### Automated Blood Sampling Surgery

Jugular cannulation was performed as previously described (41-43). Rats were anesthetized with a combination of isoflurane (100% w/w liquid vapor [Merial, UK]) and medical air. Perioperative analgesic (Carprofen; Pfizer, UK) and 2.5 mL of glucose (5%)/saline (0.9%) were administered subcutaneously. Cannulae (Scientific Commodities) were implanted in the jugular vein and exteriorized dorsally via a vascular access button attached to a spring. Springs were attached to a swivel tethering system for individual cages housed in environmentally controlled soundproof rooms under standard conditions. During postsurgical recovery, cannulae were “flushed” daily via withdrawal of blood and replaced with fresh heparinized saline (1:100) to maintain patency.

### Corticosterone Profiling

Blood samples were collected using an automated blood sampling system, with microsamples collected every 10 minutes. Plasma was separated from whole blood by centrifugation at 2000*g* at 4 °C, diluted 1 in 50 with citrate buffer, processed in triplicate and incubated overnight with 50 μL of iodine-125 (^125^I) corticosterone tracer (Institute of Isotopes, Budapest, Hungary) and 50 μL of rabbit anti-rat corticosterone antibody (kindly donated by G. Makara, Hungary). Free/bound separation was performed using charcoal dextran precipitation and centrifuged pellets. ^125^I corticosterone levels were measured using a gamma counter (Wizard-2470, Perkin Elmer, Waltham, USA). Concentrations of corticosterone (CORT) in each plasma sample were interpolated from a standard curve. Intra- and inter-assay coefficients of variation have been established as 16.65% and 13.30%, respectively.

### Adrenalectomy

Bilateral adrenalectomy was performed as described (41, 42). Rats were anesthetized with a combination of isoflurane (100% w/w liquid vapor [Merial, Bracknell, UK]) and medical air. Perioperative analgesic (Carprofen; Pfizer, Tadworth, UK), CORT (1 mg/kg, Sigma), and 2.5 mL of glucose (5%)/saline (0.9%) were administered subcutaneously. Bilateral adrenal glands were removed by the dorsal approach. After adrenalectomy, rats had ad libitum access to food and 0.9% saline throughout the experiment. ADX rats were given a subcutaneous injection with either HBC-Saline (Vehicle) or CORT-HBC-Saline (3 mg/kg CORT equivalent) at Zeitgeber time (ZT) 1. After exactly 1 hour post-injection, rats were decapitated under terminal anesthesia (inhalable isoflurane) and brains dissected.

### Methylprednisolone Treatment

Rats were randomly assigned to treatment. The methylprednisolone (MPL) group was given MPL sodium succinate (Solu-Medrol; Pharmacia Ltd, Sandwich, UK) at 1 mg/mL ad libitum in drinking water for 5 days (prolonged treatment protocol). Concentrations of MPL in drinking water (1 mg/mL) were optimized in previous experiments as the minimum dose required to reproducibly induce prolonged GR activation and suppress endogenous corticosterone (20 mg/day) (44).

## Results

### Glucocorticoids Rapidly Trigger a Wave of GR SUMOylation

**Figure 1.**
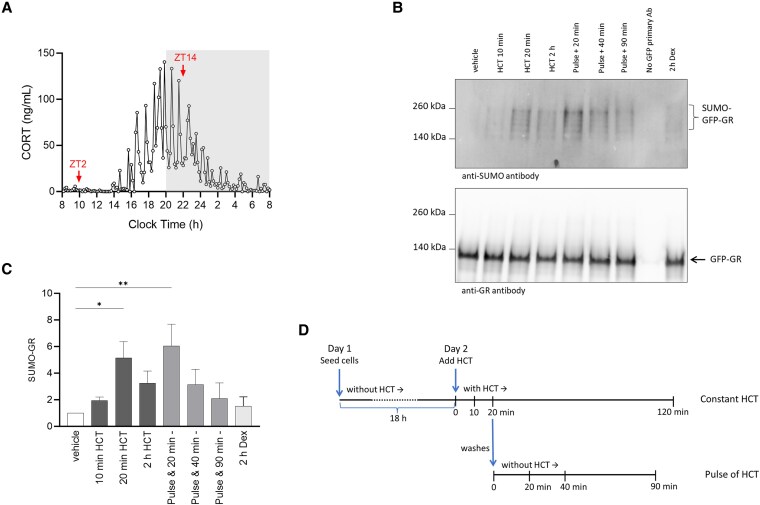
Glucocorticoids trigger a rapid wave of GR SUMOylation. (A) Endogenous 24-hour corticosterone profile of a male Lister-Hooded rat. The dark phase (20:00 to 8:00) of the light/dark cycle (12/12 hours) is shaded, and times ZT2 and ZT14, which were used for tissue collection, are indicated. (B) Representative immunoblot of GFP-Trap immunoprecipitants probed with anti-SUMO-2/3 (upper image) and re-probed with anti-GR (lower image). (C) Densitometry measurements of %SUMO-GR in GFP-Trap immunoprecipitants from GFP-GR-transfected cells treated with vehicle/HCT (50nM)/dexamethasone (100nM). Immunoblots were probed with anti-SUMO-2/3 and anti-GR. Data were normalized to vehicle-treated control. Data plotted on graph is mean ± SEM (n ≥ 3), one-way ANOVA with Dunnett's multiple comparisons test results shown on graph (*P < .05, **P < .01). (D) Experiment timeline showing constant HCT treatment and treatment with a pulse of HCT (50nM).

Endogenous GCs are secreted in pulsatile patterns into the blood (45) and remain pulsatile in extracellular fluid in rats (46) and humans (47-49). We measured corticosterone, the predominant endogenous GC in rats, using automated blood sampling every 10 minutes and show circadian (daily) and ultradian (hourly) oscillations (Fig. 1A). We hypothesized that in response to the endogenous pulsatile presentation of GCs, there might be a fluctuating pattern of GR SUMOylation. Firstly, we chose to detect SUMOylated GFP-GRα in vitro using high affinity GFP-Trap antibodies for immunoprecipitation, since SUMOylated proteins are usually present in low abundance. A549 (human lung adenocarcinoma epithelial) cells were transiently transfected with DNA plasmids encoding GFP-tagged GRα, incubated overnight in the absence of hormone, then treated with hydrocortisone (HCT, 50nM), vehicle or dexamethasone (Dex, 100nM) as a control. The GC ultradian rhythm was used as a guide to select timepoints for cell harvesting; in humans cortisol pulses are ∼16 minutes long followed by inter-pulse intervals of ∼77 minutes (50). To prevent degradation of labile SUMO moieties, SUMO inhibitor N-ethylmaleimide (NEM, 20 mM) was added during the whole cell lysis and immunoprecipitation steps. GFP-Trap immunoblots were probed with anti-SUMO-2/3 antibody first to reveal the less abundant and higher molecular weight SUMOylated GFP-GR and then re-probed with anti-GR antibody to reveal lower molecular weight unconjugated GFP-GRα.

We detected high molecular weight ladders typical of SUMOylated proteins within 10 to 20 minutes of HCT addition, indicating rapid SUMO modification of GFP-GRα (Fig. 1B). However, after 2 hours in the continuous presence of HCT (50nM) the level of GRα SUMOylation was no longer significantly elevated (Fig. 1C). To model an endogenous ultradian pulse of GCs, HCT was applied for 20 minutes, then cells were washed twice to remove hormone and incubated further in media without HCT (Fig. 1D). High levels of SUMO-conjugated GRα were detectable 20 minutes after hormone washout, but after 40 and 90 minutes in the absence of HCT GRα, the SUMOylation levels were no longer elevated. These data show that GC treatment causes a rapid, transient wave of GRα SUMOylation that peaks within minutes of GC activation and declines in the absence of GCs but also declines in the continued presence of GCs.

### GC-Induced GR SUMO Modification Promotes GR Protein Degradation

**Figure 2.**
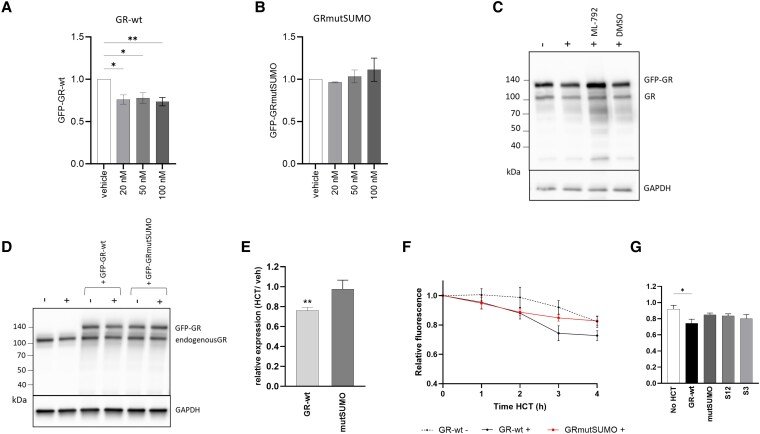
Ligand-induced GR downregulation requires GR SUMOylation. (A) HCT treatment (20/50/100nM for 6 hours) of GFP-GR-wt-transfected A549 cells decreased GFP-GR protein levels (n = 3-4); one-way ANOVA with Dunnett's multiple comparisons test results shown. (B) HCT treatment (20/50/100nM for 6 hours) of GFP-GRmutSUMO-transfected A549 cells did not decrease GFP-GRmutSUMO protein levels (n = 4; one-way ANOVA, Dunnett's multiple comparisons test results not statistically significant. (C) Western blot of A549 cells transfected with GFP-GR-wt and treated with vehicle or HCT (50nM for 6 hours); lane 3 and 4 cells were pretreated with ML792 (1µM) or vehicle (0.1% (v/v) DMSO) for 18 hours prior to HCT addition. (D) Representative Western blot showing the effect of treatment ± HCT (50nM for 6 hours) on cycloheximide-pretreated A549 cells: untransfected cells, cells transfected with GFP-GR-wt and cells transfected with GRmutSUMO. (E) Densitometry results showing GFP-GR-wt downregulation by HCT (50nM for 6 hours) in cycloheximide-pretreated cells (50 µg/mL 1 hour prior to HCT addition) was blocked by GR SUMO site mutations. Data represent GAPDH-normalized Western blot densitometry measurements of transfected GFP-GR from HCT-treated relative to vehicle-treated cells (n = 3/4); unpaired t-test results shown. (F) Cycloheximide chase assay showing GFP fluorescence assay data from A549 cells transfected GFP-GR-wt/GFP-GRmutSUMO and treated with HCT (50nM) for up to 4 hours (n = 3/4) relative to expression at time = 0. (G) The 3 hours timepoint of the cycloheximide chase assay showing GFP-GR downregulation due to HCT. A549 cells were transfected with GFP-GR constructs encoding wild-type GR, the full triple mutant (GRmutSUMO), or partial mutants with SUMO site mutations only in the N-terminal (GRmutS12) or only in the C-terminal (GRmutS3) (n = 4). Western densitometry data were normalized to GAPDH, in graphs A, B, and E. Graphs are presented as mean ± SEM. Effect of treatment *P < .05, **P < .01.

GC activation is linked with GR protein downregulation (8). We tested if GR SUMO modification was similarly linked with GR protein downregulation. HCT treatment (20/50/100nM for 6 hours) of A549 cells transiently transfected with wild-type GFP-GRα showed GFP-GRα downregulation by Western blotting as expected, even at low physiological hormone concentrations (20nM HCT) (Fig. 2A). We introduced point mutations (lysine to arginine) at each of the 3 SUMO attachment sites in the GFP-GRα expression plasmid to generate SUMO-deficient GFP-GRα (GRmutSUMO). GRmutSUMO did not show GC-induced downregulation (Fig. 2B, Fig. S2 (38)). Additionally, cells were treated with the SUMO modification blocker ML792 which inhibits the initial activation step of SUMOylation and prevents SUMO1 and SUMO2 attachment (51). The SUMO blocker ML792 increased GR levels indicating that SUMOylation is required for GC-induced GR downregulation (Fig. 2C). With these findings, we conclude that SUMO modification of GR promotes ligand-induced GR downregulation.

To test whether SUMO-dependent GR downregulation was due to reduced GR synthesis or due to increased GR protein degradation, we treated A549 cells with the translation blocker cycloheximide (50 µg/mL) to prevent new protein synthesis and reveal degradation of the existing intracellular pool of GR protein.

In the presence of cycloheximide and after 6 hours HCT treatment (50nM), SUMO-competent GFP-GR-wt showed hormone-induced downregulation by Western blotting. By contrast, SUMO-deficient GRmutSUMO was not downregulated (Fig. 2D and 2E). Using cycloheximide chase with a sensitive GFP quantification assay, we found ligand-induced degradation of GFP-GR-wt after 3 hours HCT (50nM), but no significant downregulation of GFP-GRmutSUMO after 3 hours HCT (Fig. 2F). We further investigated the effect of GR SUMO attachment site mutations by comparing the degradation of 2 partial SUMO mutants: GFP-GRmutS12 which is mutated at SUMO attachment sites S1 and S2 in the N-terminal, and GFP-GRmutS3 which is mutated at SUMO attachment site S3 in the C-terminal. In contrast to GFP-GR-wt, neither the full nor the partial SUMO site mutants showed HCT-induced degradation at the 3-hour timepoint (Fig. 2G, Fig. S3 (38)).

These experiments demonstrate that GC-induced GR degradation is impaired by GR SUMO site mutations and show that GC-induced GR SUMOylation promotes GR protein degradation.

### Glucocorticoid Withdrawal Increases Endogenous GR Protein Expression

**Figure 3.**
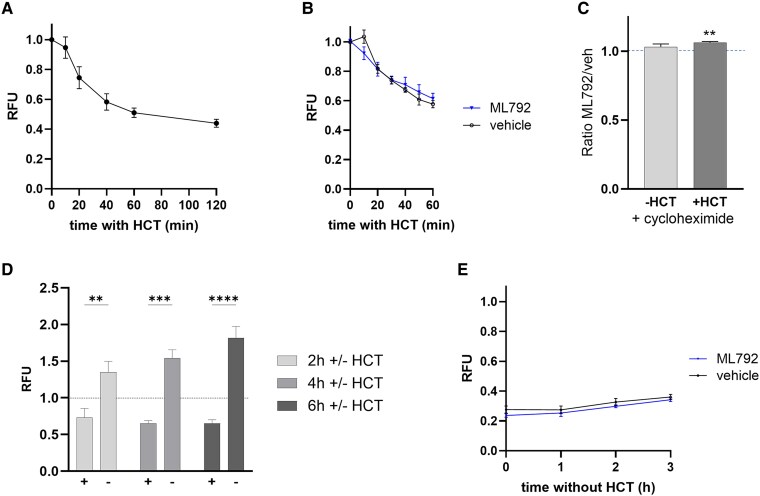
Glucocorticoid withdrawal increases endogenous GR protein expression. (A) Timecourse showing downregulation of GR-HiBiT following exposure to 50nM HCT, in GR-HiBiT A549 cells. Relative fluorescence units (RFU) represent Nano-Glo® HiBiT lytic detection assay luminescence readings after background subtraction and normalization to luminescence at t = 0 minutes. Data represent mean ± SEM, n = 3/4. (B) Timecourse showing downregulation of GR-HiBiT following pretreatment with 1µM ML792/vehicle (DMSO 0.1% [v/v]) for 1 hour and exposure to 50nM HCT, in GR-HiBiT A549. Data represent mean ± SEM, n ≥ 3. (C) Effect of ML792 (ratio ML792-treated/vehicle-treated) on GR-HiBiT expression after 1 hour HCT treatment, in GR-HiBiT A549 cells. Cells were pretreated with vehicle (DMSO 0.1% [v/v])/1 µM ML792, ± cycloheximide, for 1 hour then exposed to 50nM HCT for 1 hour. Data represent mean ± SEM, n ≥ 9, 2-way ANOVA (2 treatments) with Tukey's multiple comparisons test. (D) GR-HiBiT expression following withdrawal of hormone, in GR-HiBiT A549 cells pretreated with 50nM HCT for 18 hours. Cells were subjected to ×3 media exchanges to wash out (−) or maintain (+) HCT, then further incubation for 2/4/6 hours in the absence/presence of HCT. Data represent mean ± SEM, n = 3, 2-way ANOVA (treatment and time) with Tukey's multiple comparisons test. (E) Timecourse showing effect of ML792 on upregulation of GR-HiBiT following hormone withdrawal, in GR-HiBiT A549 cells pretreated with 50nM HCT for 18 hours. Pretreatment with 1µM ML792/vehicle (DMSO 0.1% [v/v]) for 1 hour was followed by media replacements to wash out HCT. Data represent mean ± SEM n = 3. Effect of treatment *P < .05, **P < .01, ***P < .001, ****P < .0001.

Following the experiments using transiently transfected GR constructs, we wanted to measure the effects of HCT-induced GR SUMOylation on endogenous GR expression. To this end, a CrispR-modified A549 cell line was generated in which the endogenous GR sequence was tagged by the addition of a very small 11–amino acid sequence tag HiBiT, using published donor and guide RNAs (37). The CrispR modification was confirmed by Sanger sequencing and CrispR-modified cells expressed HiBiT-labeled proteins of the expected size (94-97 kDa) (Fig. S1 (38)). Using the CrispR-modified A549 GR-HiBiT cells, it was possible to detect endogenous GRα protein in cell lysates by a sensitive bioluminescence assay in which the small HiBiT binds to a larger subunit LgBiT and produces quantitative bright bioluminescence in the presence of substrate furimazine (52). A549 GR-HiBiT cells incubated without GCs for 24 hours had high detectable luminescence, while exposure to hormone (HCT 50nM) reduced luminescence over time (Fig. 3A) in a dose-dependent manner (Fig. S4 (38)). This confirmed that we were able to sensitively measure endogenous GRα protein levels and demonstrated rapid endogenous GRα protein downregulation upon hormone exposure.

We tested whether hormone-induced GR downregulation could be impaired by SUMO inhibitor ML792. SUMO inhibition had a trend toward increased GR-HiBiT expression after ∼30 minutes HCT (Fig. 3B). We performed experiments in the presence of cycloheximide and found greater GR-HiBiT expression with SUMO inhibitor ML792, relative to vehicle treatment (Fig. 3C), that is, reduced GR degradation when SUMOylation was inhibited. This shows that a SUMOylation-dependent process enhances ligand-induced GR degradation, which is in agreement with our transient transfection results. There was no difference in cell viability between ML792 and vehicle-treated cells (Fig. S5 (38)).

Although the observed SUMO-dependent ligand-induced GR-HiBiT degradation was only a small effect, the effect could accumulate incrementally over time to a physiologically relevant level of GR depletion, if glucocorticoids are continuously present. An important question remained as to whether GR levels could be increased by the reverse process.

We found that after ligand withdrawal, endogenously controlled GR-HiBiT levels gradually increased (Fig. 3D). Cells were preincubated overnight in the presence of HCT (50nM) to ensure that GR-HiBiT levels were depleted at the start of the experiment. After 3 media changes to wash out hormone and replace media without/with HCT, the expression of GR-HiBiT began to increase after only 2 hours without hormone (Fig. 3D). This recovery of GR-HiBiT protein was not altered by ML792 treatment (Fig. 3E).

In summary, both our transiently transfected GR (GFP-GR) and endogenously regulated GR (GR-HiBiT) experiments support the conclusion that GR SUMO modification increases hormone-induced GR degradation. Importantly, endogenous GR protein expression could increase in the absence of hormone.

### Glucocorticoids Rapidly Trigger Endogenous GR SUMOylation and Ubiquitinylation

**Figure 4.**
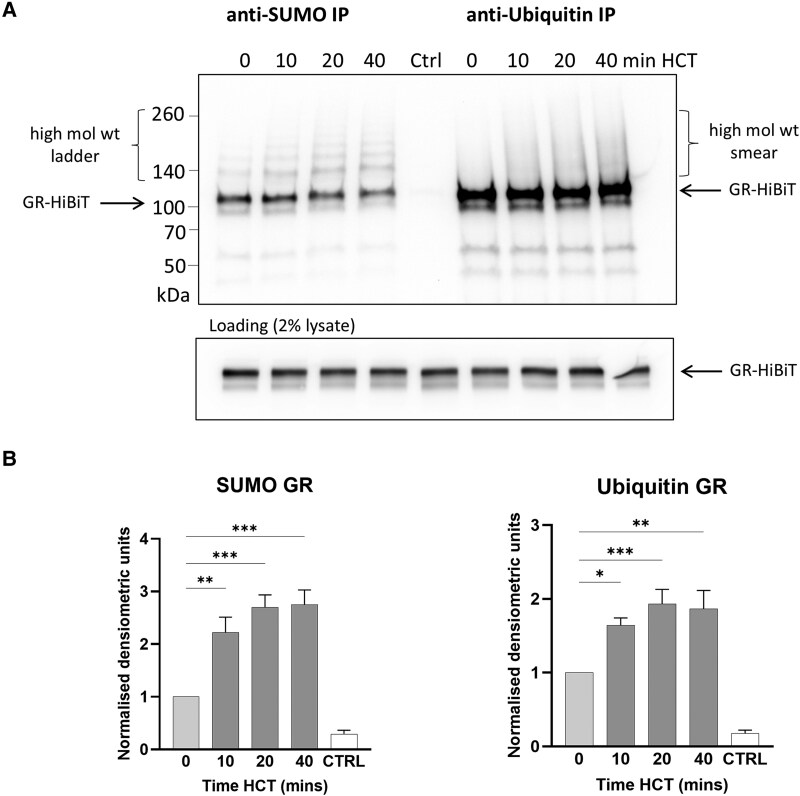
Rapid endogenous GR SUMOylation and ubiquitinylation following hormone exposure. (A) Immunoprecipitation with anti-SUMO, anti-ubiquitin, or no antibody (Crtl) magnetic agarose beads from lysates from GR-HiBiT A549 cells exposed to 50nM HCT for 0, 10, 20, 40 minutes. HiBiT blotting was used to reveal immunoprecipitated GR-HiBiT and the 2% lysate blot was probed with anti-GR antibody. (B) Densiometric measurements of GR-HiBiT immunoprecipitated by anti-SUMO/anti-ubiquitin and revealed by HiBiT blotting. Data shown represent mean ± SEM (n = 3/4) normalized to GR-HiBiT level at 0 minutes HCT treatment, one-way ANOVA.

Antibody conjugated magnetic agarose beads were used to immunoprecipitate SUMOylated and ubiquitinylated proteins from whole cell lysates of CrispR-modified A549 cells treated with hormone. Blots were probed for the HiBiT protein tag to detect endogenously regulated GR-HiBiT. The SUMO immunoprecipitations revealed high molecular weight ladders of bands representing SUMO-modified GR-HiBiT which started to increase after just 10 minutes HCT exposure (Fig. 4A and 4B). The ubiquitin immunoprecipitations showed a smear of high molecular weight products, representing ubiquitinylated GR-HiBiT, which also increased from 10 minutes HCT (Fig. 4A and 4B). Unmodified GR-HiBiT was also present, presumably as a result of GR dimer/tetramerization (53). The dynamic nature of SUMO and ubiquitin modifications is revealed by their rapid appearance within minutes. Their co-occurrence, following hormone stimulation, provides evidence for linking hormone-induced GR SUMOylation to GR degradation.

### Effects of GR SUMOylation on Transcription

We used HEK293 cells stably expressing wild-type GR (GR-wt), or SUMO-deficient GR (GR3KR) which contain lysine-to-arginine mutations at the 3 GR SUMO consensus sites (36). It has been shown that GR SUMOylation in GR3KR cells is severely defective, chromatin binding and target gene expression is altered in a gene-specific manner in response to the synthetic GC dexamethasone (100nM for 6 hours) (31). We measured heteronuclear RNA (hnRNA) expression in response to the endogenous ligand hydrocortisone (HCT 50nM) for 20 minutes or 6 hours. The short 20-minute treatment was used to look for early changes in gene transcription corresponding to rapid GR SUMOylation effects, to ensure primary transcriptional effects were captured rather than secondary regulation, and to model a physiological ultradian pulse of GCs. The hnRNA expression was measured by RT-qPCR at known GC-regulated genes: 3 GC-induced genes (FKBP5, TSC22D3, and DUSP1) and 3 GC-repressed genes (CABLES1, CCND2, SOX2) (54). Quantification by reference to a genomic DNA standard curve was used so that the absolute levels of RT products could be measured without needing comparisons with housekeeping genes whose expression could also have been altered by GR's widespread effects on genomic transcription.

**Figure 5.**
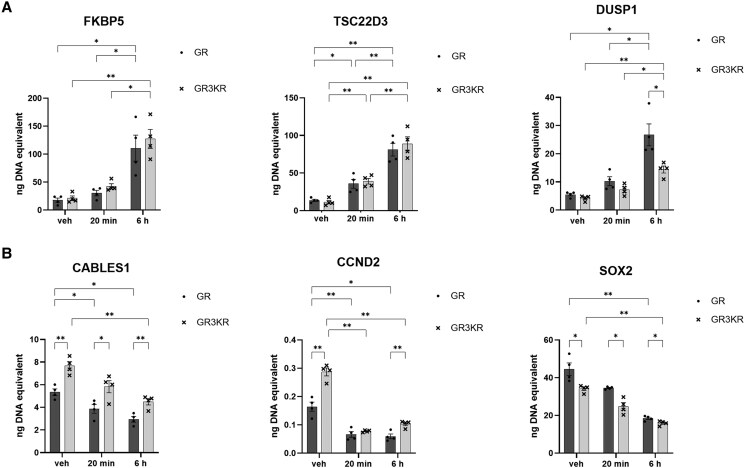
Transcriptional effects of limited GR SUMOylation. (A) Effect of vehicle/20 minutes/6 hours HCT (50nM) treatment on HEK293-GR-wt and HEK293-GR3KR cells (42). RT-qPCR measurements of known GC-induced genes: FKBP5, TSC22D3, DUSP1, by absolute quantification compared to genomic DNA amplification. (B) Effect of vehicle/20 minutes/6 hours HCT (50nM) treatment on HEK293-GR-wt and HEK293-GR3KR cells. RT-qPCR measurements of known GC-repressed genes: CABLES1, CCND2, SOX2 by absolute quantification compared to genomic DNA amplification. Data represent mean ± SEM (n = 4), 2-way ANOVA, effect of treatment *P < .05, **P < .01.

All 3 GC-induced genes tested showed hnRNA induction by both GR-wt and GR3KR after 6 hours of HCT treatment (Fig. 5A), as expected, with TSC22D3 showing a significant increase at the early 20-minute timepoint. SUMO deficiency had minimal effects on the induction of FKBP5 and TSC22D3, and reduced DUSP 1 induction at the later 6-hour HCT treatment time.

All 3 GC-repressed genes showed hnRNA repression by GR-wt and GR3KR after 6 hours HCT treatment (Fig. 5B), as expected. The transcriptional changes we observed with HCT were consistent with previously published gene-specific dexamethasone regulation in HEK293 cells (36). In contrast to the GC-induced genes, SUMO deficiency had a marked effect on all 3 GC-repressed genes we tested. We found both upregulation and downregulation effects due to SUMO deficiency; GR3KR increased CABLES1 and CCND2 hnRNA and decreased SOX2 hnRNA transcription.

It was surprising to find GR SUMO site mutations altered gene repression in the absence of hormone, because classically GR only interacts with DNA after translocating to the nucleus in the presence of ligand (55). We conclude that unliganded GR can regulate gene repression, in a SUMOylation-dependent manner.

### Endogenous GR SUMOylation Shows Circadian Fluctuations

**Figure 6.**
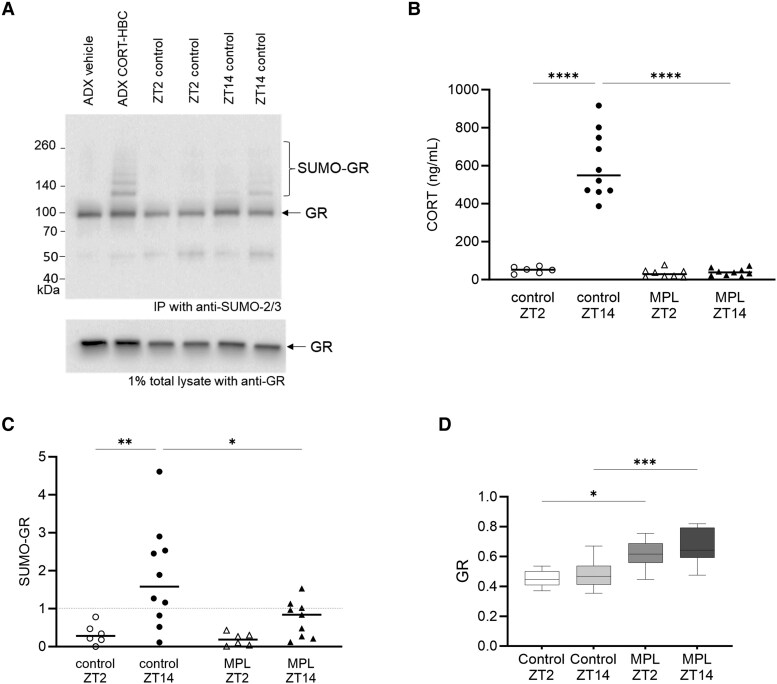
Circadian fluctuations in GR SUMOylation. (A) Representative blot showing SUMO-2/3 immunoprecipitation of prefrontal cortex tissue (PFC), from control rats at circadian time points ZT2 and ZT14 and from bilaterally adrenalectomized (ADX) control rats, probed with anti-GR and showing characteristic higher molecular weight SUMO bands. In parallel, 1% lysate samples were probed with anti-GR for calculating % SUMOylated GR. (B) CORT assay measurements from trunk blood (ng/mL) according to treatment groups. Data represent CORT levels in individual rats (mean n = 3) and mean of treatment groups (n = 6-10). Effect of treatment (*P* < .05) one-way ANOVA. Tukey's multiple comparisons test. (C) Densitometry measurements of SUMO-GR from immunoblots probed with anti-GR from control and MPL-treated rat PFC at circadian time points ZT2 and ZT14. SUMO-GR was normalized to levels in ADX rats injected subcutaneously with 3 mg/kg CORT-HBC at 1 hour prior to sacrifice. Data represent individual rat SUMO-GR levels and mean SUMO-GR levels (n = 6-10), one-way ANOVA, Tukey's multiple comparisons test. (D) Densitometry of GR level in 1% total lysates used for PFC SUMO-2/3 immunoprecipitation, probed with anti-GR. Data represent mean (n = 6-10). Effect of treatment (*P* < .05) one-way ANOVA. Tukey's multiple comparisons test. Effect of treatment *P < .05, **P < .01, ***P < .001, ****P < .0001.

Corticosterone (CORT), the predominant GC in rats, is low at the start of the rats' inactive phase (light phase) and peaks at the start of their active phase (dark phase). In order to measure in vivo GR SUMOylation, we assessed GR from rat brain tissue (prefrontal cortex, PFC) at ZT2 and ZT14. At ZT2, 2 hours after lights on, CORT levels were expected to be low and at ZT14, 2 hours after lights off, CORT levels were expected to have peaked (Fig 1A). Controls for high and low circulating CORT levels were taken at ZT2 from the PFC of ADX rats treated acutely with a bolus injection of either CORT (3 mg/kg CORT-HBC-Saline) or vehicle (HBC-Saline) respectively. Since bilateral ADX removes endogenous GCs and negative feedback on GR expression, we accordingly found elevated GR expression in PFC from ADX rats compared to untreated animals, which confirmed that the PFC is sensitive to the level of circulating GCs (Fig. 6A). GR SUMOylation was undetected in PFC from vehicle-treated ADX rats, whereas ladders of high molecular weight bands corresponding to SUMOylated GR were evident in PFC of CORT-treated ADX rats (Fig. 6A). The ADX CORT samples were used as positive controls to confirm successful extraction of SUMOylated GR and for normalization between sample groups.

In PFC from untreated rats, low GR SUMOylation levels were found at ZT2, while significantly higher GR SUMOylation levels were found at ZT14 (Fig. 6B, control). Variability at ZT14 was expected, due to presence of high amplitude short pulses of endogenous GCs at this time of day (Fig. 1A), combined with the transient nature of GR SUMOylation as demonstrated in our cell experiments (Fig. 1C). Our in vivo findings demonstrate that GR SUMOylation shows circadian oscillations, in keeping with circadian levels of endogenous GCs (Fig. 6C) and that GR SUMOylation fluctuates in normal physiology.

Prolonged treatment with the long-acting synthetic GC MPL limited GR SUMOylation at ZT14 (Fig. 6B, MPL). We also found GR protein expression was increased in these samples (Fig. 6D). It was surprising that prolonged MPL treatment caused upregulation of GR, but this probably reflects the complexity of in vivo GC regulation and negative feedback in the brain from endogenous and exogenous GCs. Within the tissue, reduced GR SUMOylation correlated with increased GR expression, corresponding with our finding that SUMOylation is linked to GR degradation. Together, these findings demonstrate that in vivo GRs in rat brain show a circadian pattern of SUMOylation which is disrupted by prolonged treatment with long-acting GCs.

## Discussion

We have demonstrated for the first time in vivo that there are SUMO modifications of the GR that show circadian variation, and that exogenous GCs can disrupt these GR SUMOylation oscillations. We also show that GCs induce transient GR SUMOylation that is linked to GR degradation, which in turn generates resistance to subsequent signal transduction and attenuates ligand responses.

Important dynamic consequences follow from the association of GC activation of GRs with GR downregulation. Degradation of GC-activated GRs ensures that activated GRs are cleared from chromatin so transcription signals are of limited temporal duration maintaining sensitivity to subsequent GC signals (9). The sensitivity of other circadian proteins may be similarly regulated by SUMO modification; BMAL (Basic Helix-Loop-Helix ARNT Like 1) is SUMO-regulated and simultaneously promotes transactivation and ubiquitin-dependent degradation (56), while SUMO1 and SUMO2 modification of PER2 (Period Circadian Regulator 2) has been shown to have differential effects on PER2 turnover and on transcriptional suppression (57).

Receptor degradation following ligand activation creates a self-limiting protection from excessive GC signaling. Pulsatile GC release, which occurs in normal physiology with circadian and ultradian patterns of GC pulse peaks and nadirs, provides a mechanism that can avoid the problem of chronic signal exposure leading to receptor downregulation; which is problematic in GC therapy (6). Pulsatile GC signals provide windows of time during which GRs are not activated and therefore not downregulated, allowing the level of GR protein expression to be restored. Pulsatile GC peaks transmit signals, while pulsatile hormone troughs promote recovery of receptor expression and signal responsivity by avoiding chronic receptor degradation. Our A549 cell data demonstrate that GR levels start to recover within 2 hours in the absence of GCs, suggesting that the circadian nadir is long enough for GR protein expression to be substantially restored, even after significant receptor depletion. Although ultradian nadirs are of shorter duration, both ultradian and circadian GC nadirs are likely to contribute to maintaining GR expression levels throughout the 24-hour cycle by providing low-hormone windows. Interestingly, the first pulse of GCs at the start of the active phase will meet with the highest level of GR expression and therefore be particularly effectively transduced into transcriptional responses, which may be advantageous in priming cells and tissues for the demands of active phase. Many hormones including pituitary, gluco- and mineralocorticoid, catecholamine, gonadal sex steroid, parathormone, insulin and glucagon are pulsatile with episodic secretion (58). Hormone pulsatility appears to be a common homeostatic mechanism for maintaining receptor expression and together with signal responsivity over time.

Disruption of the close coupling between GC activation and GR downregulation dysregulates GC responses, for example by limited GR SUMOylation. At GC-induced sites, SUMO-deficient GR has been shown to increase chromatin binding, opening, and gene expression (31, 32). Our findings add that GR SUMO deficiency impairs GR degradation, which is consistent with the reported longer chromatin residence times of SUMO-deficient GR (31) and potentiated transcriptional activity (32).

We found that GR SUMOylation had transcriptional effects in the absence of ligand, revealing a role for unliganded GR in gene regulation. This surprising finding warrants further investigation, which is beyond the scope of this study. While it is theoretically possible for nuclear unliganded GR to interact indirectly with chromatin, it is more probable that cytoplasmic unliganded GR influences gene expression via a nongenomic pathway. Indeed, a cytoplasmic unliganded GR interaction of this type has recently been described in which unliganded GR and RAS interact to restrain RAS activation, while loss of GR or ligand activation unleashes RAS activation (59). Whatever the molecular pathway by which unliganded GRs regulate gene expression, the finding of a regulatory role for unliganded GR highlights the importance exogenous GC dosing, timing, and the need to study physiological GC stimulation. Supraphysiological doses of potent synthetic GCs like dexamethasone, which have been widely used in previous GC studies, are likely to obscure unliganded GR effects.

Global increases in SUMOylation occur in response to cell stresses, from hypoxia to nutrient stress (20). Tissue-specific differences in the SUMO equilibrium (SUMO modification/deconjugation) and SUMO dynamics have also been reported, such that some tissues (eg, liver) have higher basal levels of SUMOylated proteins, while other tissues (eg, brain) have more free SUMO available for conjugation (60). GR SUMOylation is enhanced by RSUME which has tissue-specific expression and is induced under stress conditions (61). This suggests that some brain regions, where abundant provision of free mature SUMO is in combination with high expression of SUMO enhancers (eg, RSUME), could have high responsivity and be capable of robust SUMO-dependent stress-induced responses. Since GR transcriptional effects depend on how readily GR is SUMOylated (36), increases in GR SUMOylation provide a connection between cell stress and inflammatory responses. Interestingly, our in vivo data showed reduced GR SUMOylation in MPL-treated rat PFC, demonstrating that synthetic GCs are capable of limiting GR SUMOylation in brain tissue. More studies are needed to understand the consequences of decreased GR SUMOylation in the context of GC feedback regulation.

**Figure 7.**
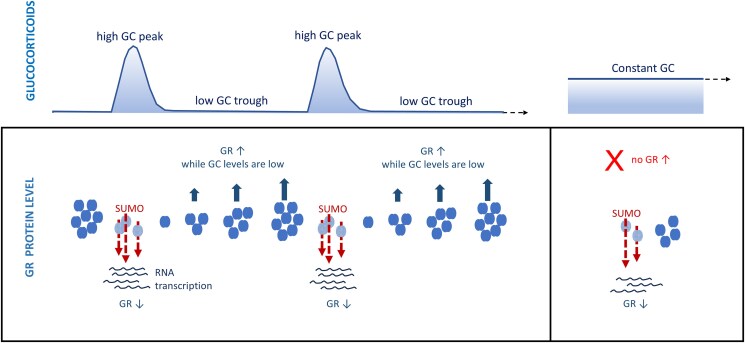
Model depicting how glucocorticoid oscillations dynamically change glucocorticoid sensitivity by altering GR expression.

Our data predict that the ultradian and circadian fluctuations of GCs in normal physiology trigger fluctuations in GR SUMOylation, GR ubiquitinylation, and fluctuations in GC responsivity due to rising and falling GR expression (Fig. 7). Endogenous oscillations of GCs trigger waves of GR activation which can be observed as oscillating levels of nuclear GR, detected by nuclear fractionation (62). GC-activated nuclear GR engages with chromatin and regulates gene transcription, both of which show oscillating patterns (34, 41, 63-65). While GC levels are low, GR levels rise which increases GC sensitivity; in this way glucocorticoid oscillations dynamically change GC responsivity by altering GR expression (Fig. 7). Ultradian fluctuations in GC responsivity have been reported in rats, as the magnitude of ACTH and CORT responses to a stressor depended critically on the timing of stress with respect to endogenous CORT oscillations (66). By contrast, pathological chronic inflammatory stress is associated with reduced GC responsivity and a dysregulated circadian rhythmicity, for example in male Piebald-Viral-Glaxo (PVG) rats with induced arthritis (67).

Current GC therapies chronically expose tissues to long-lasting GCs which limit the GRα protein pool by promoting ongoing receptor degradation, especially during periods when endogenous hormone levels are low and when GRα levels should be recovering. The finding that GRα expression recovers in the absence of GCs suggests new therapeutic possibilities. Pharmacological inhibition of GC activity during the circadian GC nadir could increase GRα expression and GC sensitivity. Short half-life inhibitors and asymmetric dosing regimens would be needed to permit GC signaling during the active hours and restrain GC activity overnight. Alternatively, the identification of GR SUMOylation as a modulator of GR downregulation raises the possibility of specifically targeting GRα SUMOylation to increase GRα expression. SUMOylation is emerging as a potential novel drug target: therapeutic enhancement of SUMOylation has achieved remarkable clinical responses, for example in acute promyelocytic leukemia where arsenic causes SUMOylation of the driver oncoprotein (68), and SUMOylation inhibitors are currently under clinical trials for anti-leukemic effects (69). Interestingly, the lowest levels of many hormones coincide overnight (∼12 to 3 Am) in healthy individuals (70) suggesting that dynamic SUMOylation inhibition targeted to this time window could be well tolerated.

To conclude, we show that GC activation triggers GRα SUMOylation which increases GRα degradation. This creates a conflict between GC signal transduction and maintaining GRα expression, that is resolved by endogenous oscillating GC hormone patterns. We show that GC nadirs have an important function in maintaining GRα expression over time, emphasizing the importance of hormone presentation patterns for good signaling responses. Our data suggests that periodic reductions in GC levels could restrain GRα degradation, increase GRα expression and promote GC sensitivity.

## Data Availability

Some or all datasets generated during and/or analyzed during the current study are not publicly available but are available from the corresponding author on reasonable request.

## Abbreviations

ADX: adrenalectomized
CORT: corticosterone
Dex: dexamethasone
DMEM: Dulbecco's Modified Eagle Medium
GC: glucocorticoid
GR: glucocorticoid receptor
GR3KR: SUMO-deficient glucocorticoid receptor
GR-wt: SUMO-competent wild-type glucocorticoid receptor
HCT: hydrocortisone
hnRNA: heterogeneous nuclear RNA
MPL: methylprednisolone
NEM: N-ethylmaleimide
PCR: polymerase chain reaction
PFC: prefrontal cortex
RSUME: RWD-containing SUMOylation enhancer
